# Interrogating the Whole-Genome Shotgun Sequence of Escherichia coli Sequence Type 127 Strain 1538RHQ, Which Harbors Virulent Antigenic Factors, Isolated from a Mastitic Cow

**DOI:** 10.1128/MRA.01057-19

**Published:** 2019-11-27

**Authors:** A. Bashir, Z. Zunita, F. F. A. Jesse, S. Z. Ramanoon, M. L. Mohd-Azmi

**Affiliations:** a Faculty of Veterinary Medicine, Universiti Putra Malaysia, Serdang, Selangor, Malaysia; b Department of Biological Sciences, Sule Lamido University, Kafin Hausa, Jigawa State, Nigeria; Broad Institute

## Abstract

We report the whole-genome sequence of Escherichia coli sequence type 127 (ST127) strain 1538RHQ, recovered from a mastitic cow in a dairy herd in Selangor, Malaysia. The objective of this study was to identify the antigenic and virulence properties that can be used as suitable targets for vaccine development against bovine mastitis.

## ANNOUNCEMENT

Escherichia coli has been reported as a common cause of bovine mastitis worldwide (1, 2). Bovine mastitis significantly affects both the economy and animal welfare (3). Infections of the mammary gland due to E. coli are usually of short duration and result in conditions ranging from mild to severe (4). Virulence genes associated with E. coli strains implicated in bovine mastitis have been studied. These include genes encoding hemagglutinin, aerobactin, P fimbria, enterohemolysin, intimin, and autoagglutinating adhesion proteins (5). This could explain the ability of E. coli to colonize many different host tissues and cause various diseases in many animals.

Here, the E. coli 1538RHQ sequence type 127 (ST127) genome was sequenced using an Illumina MiSeq 2000 instrument with the view of studying its virulence features and identifying candidate vaccine targets. This isolate was obtained from a mastitic cow at a dairy farm in Selangor, Malaysia. Culture isolation, identification, and genomic DNA extraction were performed using standard procedures as previously described (6). Briefly, E. coli ST127 strain 1538RHQ was isolated from a milk sample inoculated on both blood agar and McConkey agar (Oxoid, Hampshire, England). Plates were incubated aerobically at 37°C for 24 hours, after which they were examined for growth and colony morphology. Identification was performed using the analytical profile index (API) kit (bioMérieux, France) according to the manufacturer’s instructions.

A Wizard genomic DNA extraction kit (Promega, Wisconsin, USA) was used for genomic DNA extraction based on the manufacturer’s instructions. The quality of the extracted DNA was confirmed using a NanoDrop 2000c spectrophotometer (Thermo Fisher Scientific, Waltham, MA, USA) and 1% agarose gel electrophoresis. On sequencing, a genomic library was constructed using a Nextera XT DNA library preparation kit (Illumina, San Diego, CA, USA). Sequencing was performed on an Illumina MiSeq 2000 platform with paired-end chemistry. A total of 411.7 Mb (2 × 151 bp) was obtained. After quality control was performed with BBDuk tools version 36 (http://jgi.doe.gov/data-and-tools/bbtools/), the sequence reads were *de novo* assembled using SPAdes version 3.9.0 with the careful option, which reduces the number of mismatched and short indels (kmer sizes of 21, 33, 55, and 67 and coverage cutoff turned off) (7). The genome was assembled into a total of 167 contigs with an *N*_50_ value of 145,187 bp and a largest contig size of 416,566 bp. The genome is approximately 5.2 Mb with a G+C content of 50.4%. The average coverage depth was 87.61×.

The genome was annotated using GeneMarkS+ version 4.5 (8) through the NCBI annotation pipeline. A total of 5,113 protein-coding genes, 78 tRNA operons, and 4 rRNA operons were detected. Functions of the protein-coding genes were determined from their detected sequences using BLASTN version 2.2.31+ on the NCBI database (9) and HMMER version 3.0 (10). Various virulence factors have been unveiled using the virulence factors database (http://www.mgc.ac.cn/VFs) (11). The virulence factors identified include adhesin proteins (associated with P fimbriae and type 1 fimbriae), K capsule proteins, hemolysins, aerobactin system-associated proteins, and many types of peptide permeases.

### Data availability.

This whole-genome shotgun project has been deposited at DDBJ/ENA/GenBank under the GenBank accession number QLZB00000000. The version described in this paper is the first version, QLZB01000000. The SRA data can be found under the accession number PRJNA476666.

## References

[1] Bradley AJ, Green MJ 2000 A study of the incidence and significance of intramammary enterobacterial infections acquired during the dry period. J Dairy Sci 83:1957–1965. doi:10.3168/jds.S0022-0302(00)75072-7. 11003224

[2] Talbot BG, Lacasse P 2005 Progress in the development of mastitis vaccines. Livestock Prod Sci 98:101–113. doi:10.1016/j.livprodsci.2005.10.018.

[3] Capra E, Cremonesi P, Pietrelli A, Puccio S, Luini M, Stella A, Castiglioni B 2017 Genomic and transcriptomic comparison between Staphylococcus aureus strains associated with high and low within herd prevalence of intra-mammary infection. BMC Microbiol 17:1–16. doi:10.1186/s12866-017-0931-8. 28103794 PMC5247818

[4] Zhang D, Zhang Z, Huang C, Gao X, Wang Z, Liu Y, Tian C, Hong W, Niu S, Liu M 2018 The phylogenetic group, antimicrobial susceptibility, and virulence genes of Escherichia coli from clinical bovine mastitis. J Dairy Sci 101:572–580. doi:10.3168/jds.2017-13159. 29055550

[5] Johnson TJ, Wannemuehler Y, Johnson SJ, Stell AL, Doetkott C, Johnson JR, Kim SK, Spanjaard L, Nolan LK 2008 Comparison of extraintestinal pathogenic Escherichia coli strains from human and avian sources reveals a mixed subset representing potential zoonotic pathogens. Appl Environ Microbiol 74:7043–7050. doi:10.1128/AEM.01395-08. 18820066 PMC2583479

[6] Bashir A, Zunita Z, Jesse FFA, Ramanoon SZ, Mohd-Azmi ML 2019 Whole-genome shotgun sequence of Streptococcus agalactiae sequence type 176 strain 3966RFQB from a dairy herd in Selangor, Malaysia. Microbiol Resour Announc 8:e01618-18. doi:10.1128/MRA.01618-18. 30746526 PMC6368661

[7] Bankevich A, Nurk S, Antipov D, Gurevich AA, Dvorkin M, Kulikov AS, Lesin VM, Nikolenko SI, Pham S, Prjibelski AD, Pyshkin AV, Sirotkin AV, Vyahhi N, Tesler G, Alekseyev MA, Pevzner PA 2012 SPAdes: a new genome assembly algorithm and its applications to single-cell sequencing. J Comput Biol 19:455–477. doi:10.1089/cmb.2012.0021. 22506599 PMC3342519

[8] Besemer J, Lomsadze A, Borodovsky M 2001 GeneMarkS: a self-training method for prediction of gene starts in microbial genomes. Implications for finding sequence motifs in regulatory regions. Nucleic Acids Res 29:2607–2618. doi:10.1093/nar/29.12.2607. 11410670 PMC55746

[9] Camacho C, Coulouris G, Avagyan V, Ma N, Papadopoulos J, Bealer K, Madden TL 2009 BLAST+: architecture and applications. BMC Bioinformatics 10:421. doi:10.1186/1471-2105-10-421. 20003500 PMC2803857

[10] Eddy SR 1998 Profile hidden Markov models. Bioinformatics 14:755–763. doi:10.1093/bioinformatics/14.9.755. 9918945

[11] Chen L, Yang J, Yu J, Yao Z, Sun L, Shen Y, Jin Q 2005 VFDB: a reference database for bacterial virulence factors. Nucleic Acids Res 33:D325–D328. doi:10.1093/nar/gki008. 15608208 PMC539962

